# Free-breathing T2* mapping for MR myocardial iron assessment at 3 T

**DOI:** 10.1186/s41747-020-00156-3

**Published:** 2020-04-17

**Authors:** E. E. Nazarova, G. V. Tereshchenko, D. A. Kupriyanov, N. S. Smetanina, G. A. Novichkova

**Affiliations:** 1 Radiology department, Dmitry Rogachev National Medical Research Center of Pediatric Hematology, Oncology and Immunology, Samory Mashela st., 1, Moscow, Russia 117997; 2 Philips Healthcare, Moscow, Russia; 3 3Pirogov Russian National Research Medical University, Moscow, Russia

**Keywords:** Artefacts, Heart, Iron overload, Magnetic resonance imaging, Myocardium

## Abstract

**Background:**

Timely diagnosis of cardiac iron overload is important for children with transfusion-dependent anaemias and requires modern measure methods. Nowadays, myocardial iron quantification is performed by magnetic resonance (MR) breath-hold techniques, sensitive to respiratory motion and unfeasible in patients who are unable to hold their breath. Free-breathing T2* mapping sequences would allow to scan children who cannot hold their breath for a specified duration. Our aim was to test a free-breathing T2* mapping sequence, based on motion correction by multiple signal accumulation technique.

**Methods:**

We used an electrocardiographically gated T2* mapping sequence based on multiple gradient echo at 3-T in 37 paediatric patients with haematologic disorders aged from 2 to 16. We compared T2* values of myocardium and signal-to-noise ratio of this new sequence with standard breath-holding T2* mapping sequence. T2* values were measured in the interventricular septum for both methods in studies with adequate image quality.

**Results:**

All children were scanned without complications. Five patients were excluded from analysis because of the presence of respiratory artefacts on the T2* images with breath-holding technique due to patient’s inability to hold their breath. Breath-holding T2* was 19.5 ± 7.7 ms (mean ± standard deviation), free-breathing T2* was 19.4 ± 7.6 ms, with positive correlation (*r* = 0.99, *R*^2^ = 0.98; *p* < 0.001). The free-breathing sequence had a higher signal-to-noise ratio (median 212.8, interquartile range 148.5–566.5) than the breath-holding sequence (112.6, 71.1–334.1) (*p* = 0.03).

**Conclusion:**

A free-breathing sequence provided accurate measurement of myocardial T2* values in children.

## Key points

- A free-breathing motion corrected by multiple signal accumulation magnetic resonance (MR) protocol was compared to a breath-holding protocol at 3-T in 37 paediatric patients.
- The free-breathing multi turbo field-echo sequence resulted to be less sensitive to the breath motion and provided a higher signal-to-noise ratio than the standard breath-holding technique
- T2* mapping free-breathing technique makes the MR study less depended on the ability of patients to hold their breath and may pave the way for T2* myocardial quantification in children performing cardiac MR without the anaesthetic support.

## Background

Iron overload is a condition caused by excessive accumulation of iron in the body and relates to hereditary as well as acquired hemochromatosis due to the repeated blood transfusions that become necessary in such conditions as congenital dyserythropoietic anaemia, thalassemia, sickle cell anaemia, aplastic anaemia and myelodysplasia [1]. One of the most important problems associated with iron overload is the deposition of iron in the heart resulting in a higher risk of heart failure development [2]. At early stages, iron deposition in the heart does not have a toxic effect because of the ability of ferritin-hemosiderin to buffer systems of the myocyte to maintain a low level of toxic labile cell iron [3]. However, over the time and with the saturation of the buffer system, the iron accumulation in the heart increases and leads to cardiomyopathy, which then presents with heart failure [4], this being the leading cause of premature death in patients with β-thalassemia [5]. With a timely chelation therapy, iron overload-related cardiomyopathy is reversible [6]. Usually, the manifestation of cardiovascular complications occurs after the diagnosis of severe myocardium iron overload, when the prognosis is already negative [7]. Thus, these patients need an ongoing monitoring of the iron content in the body for and early detection of myocardial iron overload for a better decision-making on therapy finally increasing the lifetime expectancy.

Until recently, the accumulations of iron in the body were estimated indirectly using serum ferritin level tests or by direct method such as liver biopsy with mass spectrometry of the samples [8, 9]. However, inflammation, ascorbate deficiency and liver function are often affecting the accuracy of serum ferritin level test results, which significantly limits its diagnostic value. In addition, the procedure of liver biopsy has a risk of internal bleedings, tissue damage and infections [10]. Of note, there is no correlation between iron deposition in the liver and in the myocardium, due to a slower iron accumulation in the heart than in the liver [11].

Tissue Doppler echocardiography could help to provide an indirect measure of subclinical cardiac involvement in these patients. However, it does not allow the direct measurements of myocardial iron concentration [12]. Endomyocardial biopsy can be used for the iron assessment in the myocardium, but the risk of complications such as pneumothorax, haemothorax, dysrhythmia, heart block and perforation are much higher in children than in adults, especially in patients with cardiomyopathy [13, 14]. Finally, the ejection fraction monitoring does not allow to detect the concentration of iron in the myocardium accurately [15].

The clinical need for a non-invasive and reliable technique for the iron content myocardium measurement led to the use of magnetic resonance (MR) T2*-weighted pulse sequences. In 2001, Anderson et al. [16] firstly described the application of a specialised gradient-echo (GRE) sequence based on the T2* relaxation phenomenon enabling the detection of magnetic field inhomogeneities. The detection of iron deposits on T2* mapping sequences is possible thanks to the ferromagnetic properties of haemosiderin, which causes a local shortening of this relaxation time and leads to the loss of MR signal intensity in the heart tissue. Currently, cardiac MR is the non-invasive reference standard for the diagnosis of excessive iron accumulation in organs and tissues [17–21]. Cardiac MR T2* mapping is based on measuring the T2* value in a region of interest (ROI) in the interventricular septum, and researches have shown that measuring T2* values in ROI of the interventricular septum significantly correlates with the iron content in the whole myocardium [7].

Electrocardiography (ECG)-gated multi-phase GRE sequences are widely used for iron assessment in the heart, but this technique requires the patients to hold their breath in order to avoid artefacts from respiratory movements [22].

We expect that cardiac MR T2* free-breathing technique described in this article paves the way towards scanning most children with transfusion-dependent anaemias from 5 to 7 years of age without the need of anaesthetic support. MR scanning technique with breathing artefacts compensation was introduced in 2015 by Kellman et al. using a 1.5-T magnet in a sample of adult patients [23]. However, in clinical practice, 3-T scanners are also widely used and needed a free-breathing T2* mapping technique testing. Thus, the aim of our study was to present the myocardium iron detection free-breathing technique based on multiple signal accumulations for the respiratory motion and motor activity corrections in children using a 3-T MR scanner.

## Methods

### Study design and study group

This was a prospective single-institution study performed in the Dmitry Rogachev National Medical Research Center of Pediatric Hematology, Oncology and Immunology during the period from 2017 to 2018. MR iron assessment examination, including cardiac T2* mapping, was conducted in all paediatric patients in accordance with the inclusion criteria of the study. Patients were scanned by both new and standard protocols to compare the image quality and resulting T2* values as well as to estimate the efficiency of the new sequence.

Inclusion criteria were patients with transfusion-dependent anaemia under 18 years of age and with more than 10 red blood cell transfusions in the past medical history. Exclusion criteria were absolute contraindications to MR (such as unsafe pacemaker or ferromagnetic implants, etc.) and the refusal to sign informed consent. Fourteen out of 17 children were scanned under general anaesthesia in the group of patients from 2 to 7 years old. Three out of 17 children were scanned without anaesthetic support due to the parent’s refusal to apply anaesthesia for the MR investigation. Sevoflurane inhalation was used for the induction and maintenance of general anaesthesia. Rocuronium bromide injection was used as a muscle relaxant to achieve breath-holding during MR study. Drug dosage was adjusted according to the age and weight of each patient. Children over 7 years old were scanned without anaesthetic support.

The local ethical committee of the Dmitry Rogachev National Medical Research Center of Pediatric Hematology approved the study. Informed consent was received from all parents of patients.

### Scan protocol

All patients underwent myocardial examination on a 3-T Achieva dStream system (Philips Healthcare, Best, The Netherlands), using a 16-channel body coil. Scanning was performed using a multi-phase fast GRE sequence, which provides multiple echoes and allows to get cardiac short-axis projections with standard parameters for either breath-holding or free-breathing. In the latter case, respiratory motion activity is compensated by averaging of multiple acquisitions to reduce error of phase encoding. Scan parameters are presented in Table 1. For both techniques we used the ECG gating.

**Table 1:** Imaging parameters for breath-holding (BH-mTFE) and free-breathing (FB-mTFE) sequences

	BH-mTFE	FB-mTFE
Number of slices	1	1
Acquisition matrix	124 × 119	124 × 122
Resolution (mm)	2.4 × 2.5 × 8	2.4 × 2.4 × 8
Field of view (mm)	297 × 297	297 × 292
Slice thickness (mm)	8	8
k-space sampling	Cartesian	Cartesian
Flip angle (degree)	25	25
Echo time (ms)	1.3	1.3
∆ Echo time (ms)	1.2	1.2
Repetition time (ms)	21	22
Number of excitations	1	8
Electrocardiography-gating	Cardiac triggered	Cardiac triggered
Respiration	Breath hold ≈ 15 sec	Respiratory trigger
Duration	12 s	2 min, 11 s

### Image analysis

**Fig. 1:**
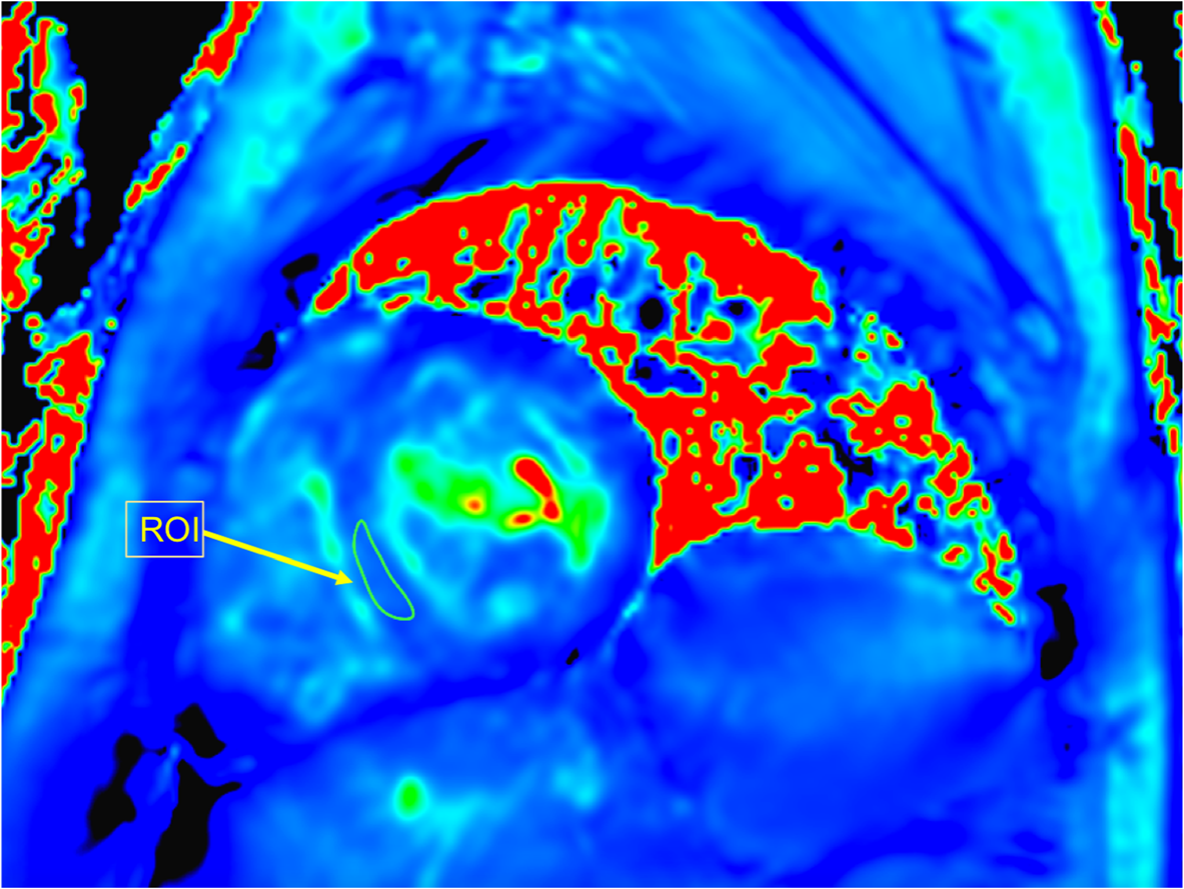
T2* map with a region of interest (ROI) placed in the interventricular septum

The T2* maps were generated automatically using the MR console (Philips Healthcare, Best, The Netherlands). The mean T2* values were measured within the equal size elliptical ROIs in the interventricular septum for both acquisition techniques (Fig. 1). To estimate the inter-reader reproducibility, two radiologists with 5 and 15 years of experience in cardiac imaging independently evaluated data of each patient. The two readers performed at least ten measurements for each patient. In 2 weeks, the same data was analysed once again by the two readers to evaluate the intra-reader reproducibility.

Image quality was assessed by measuring the signal-to-noise ratio (SNR) for one ROI in the middle of the interventricular septum and another one in the area of background noise [24, 25]. SNR was calculated as follows:

$$ \mathrm{SNR}=\frac{\mathrm{SI}\left(\mathrm{ivs}\right)}{\mathrm{SD}\left(\mathrm{air}\right)}, $$

where SI (ivs) is the value of the signal intensity measured in the drawn ROI in the interventricular septum, and SD (air) is the standard deviation of the background noise.

### Statistical analysis

STATISTICA 8.1. (StatSoft, Tulsa, OK, USA) and Office Excel 2018 (Microsoft, Redmond, WA, USA) were used. Mean T2* value and SD were calculated for each ROI. The mean value for each case was used as the optimal T2* value while the SD was used as a measure of uncertainty. For patients, except those excluded from analysis, a scatterplot between T2* values were obtained. For the inter-reader reproducibility estimate, the results were analysed by Bland-Altman analysis. For the intra-reader reproducibility estimate, the coefficients of variation were obtained. To determine the normality of the T2* values distribution and SNR results, the Shapiro-Wilk test and the frequency distribution histogram were used. For non-normally distributed data, the pairwise Wilcoxon rank sum test was used. For normally distributed data, pairwise Student *t* test and Pearson correlation were used. The threshold for significance was set at 0.05.

## Results

The study was performed in 37 patients aged from 2 to 16 years old (median age—8 years) who were under medical treatment at the Dmitry Rogachev National Medical Research Center of Pediatric Hematology, Oncology and Immunology, diagnosed with β-thalassemia (*n* = 14), Diamond–Blackfan anaemia (*n* = 13), dyserythropoietic anaemia 1 (*n* = 5) and acquired aplastic anaemia (*n* = 5). They were 11 females and 26 males (ratio 1:2.4).

All patients successfully completed the MR study without complications. The children from 2 to 7 years of age (14 of 37 patients) underwent the scanning using general anaesthesia.

**Fig. 2:**
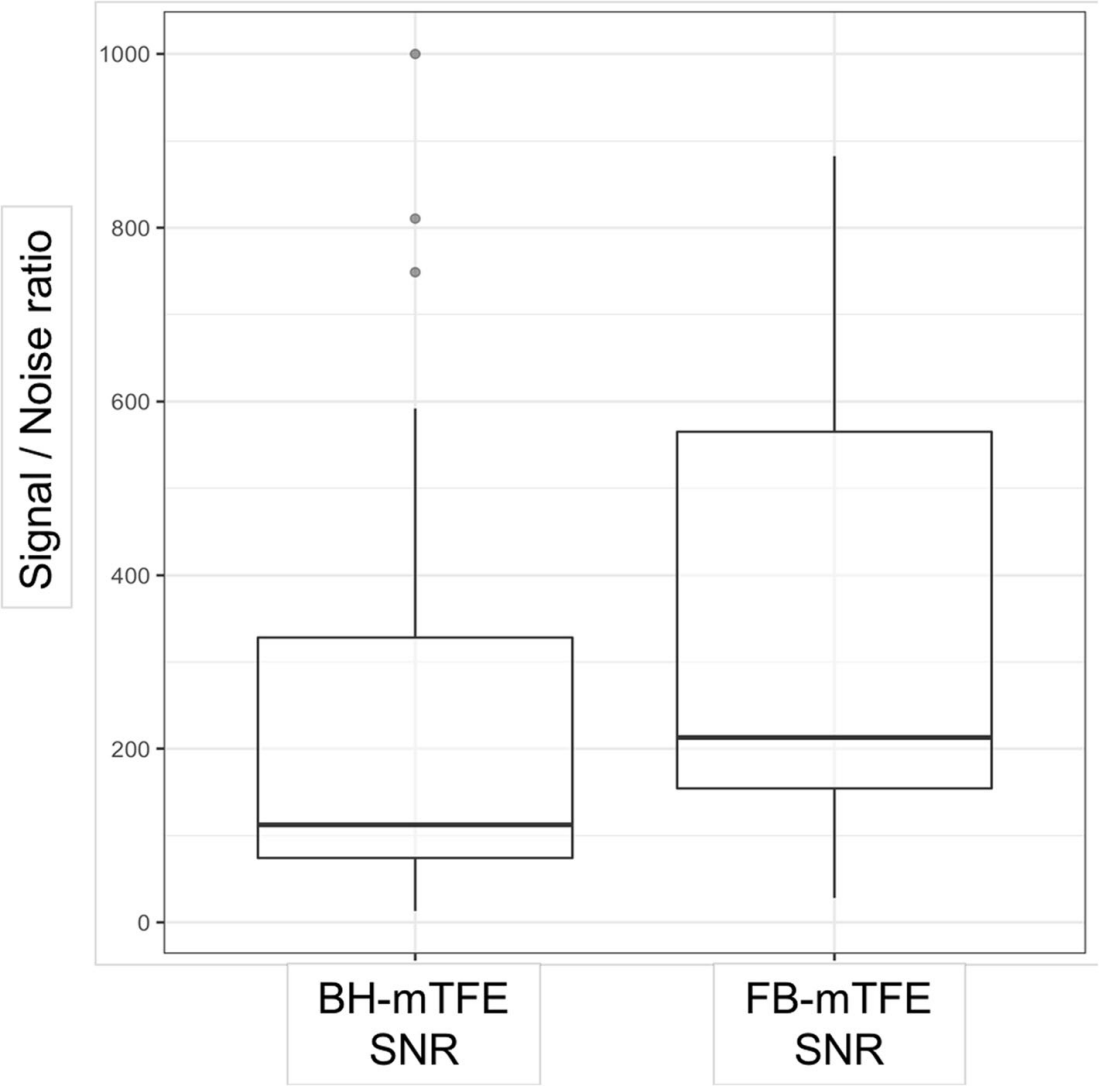
Boxplots of the signal-to-noise ratio (SNR) for breath-holding (BH-mTFE) and free-breathing (FB-mTFE) sequences. SNR of BH-mTFE = 112.6, interquartile range 71.1−334.1. SNR of FB-MTFE = 212.8; interquartile range 148.5−566.5. • Outliers

The results of Shapiro-Wilk test for the SNR of both BH-mTFE sequence and FB-mTFE sequence showed a non-normal distribution (*p* = 0.00001 and *p* = 0.001 respectively). Therefore, the pairwise Wilcoxon rank sum test was used. Wilcoxon rank sum test showed that the SNR of the FB-mTFE sequence was significantly higher (median 212.8, interquartile range 148.5–566.5) than that of the BH-mTFE (median 112.6, interquartile range 71.1–334.1) (*p* = 0.003) (Fig. 2).

**Fig. 3:**
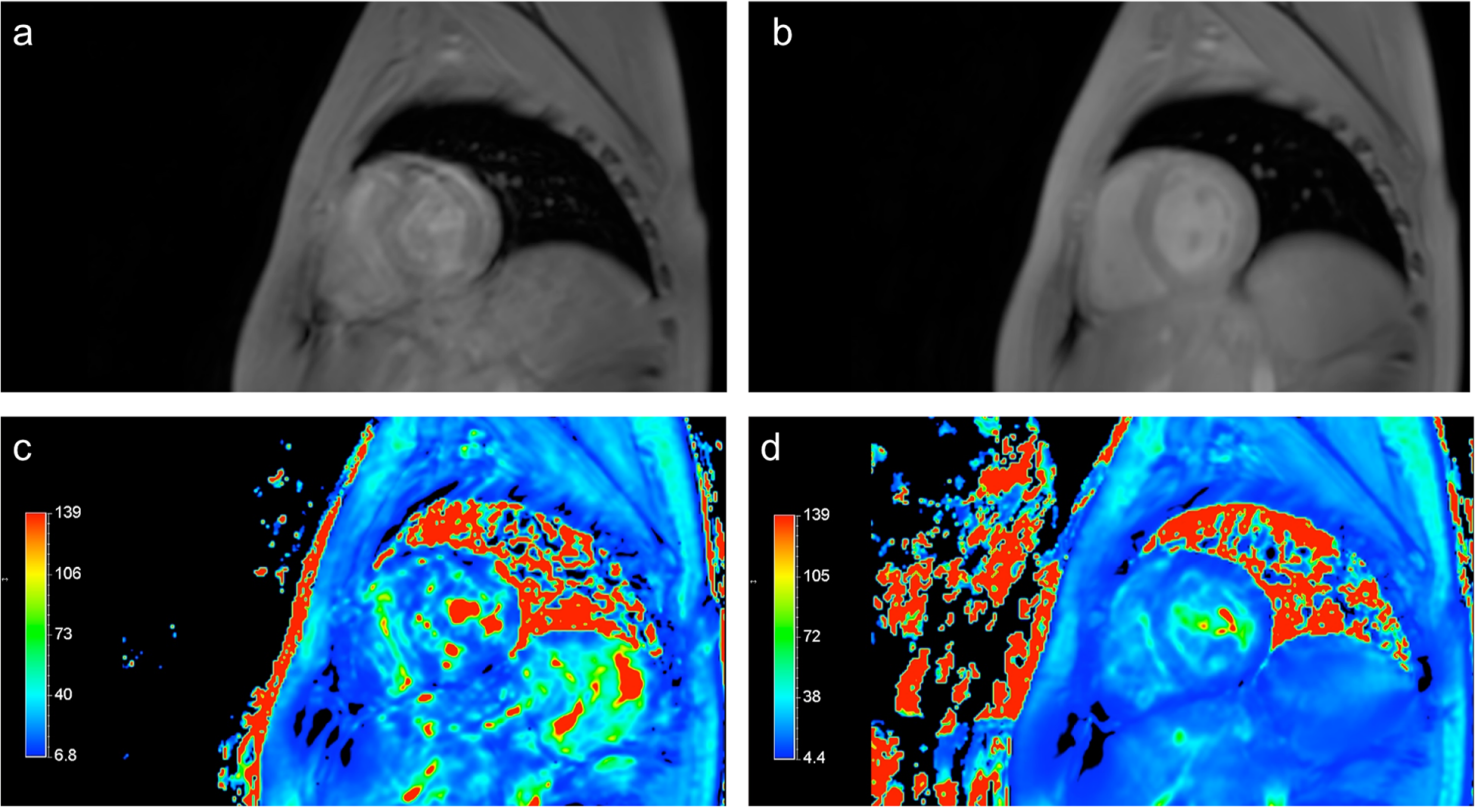
Example of data excluded from analysis regarding a 5-year old boy with Diamond–Blackfan anaemia who underwent the cardiac magnetic resonance without anaesthetic support. **a** Original image obtained using the breath-holding technique. **b** Original image obtained using the free-breathing technique. **c** T2* map obtained using the breath-holding technique. **d** T2* map obtained using the free-breathing technique

Five of 37 studies (14%) were excluded from analysis because of insufficient image quality due to the inability of patients to hold their breath. Three of the excluded patients from 5 to 7 years of age with the diagnoses of the dyserythropoietic anaemia (*n* = 1) or Diamond–Blackfan anaemia (*n* = 2) were scanned without anaesthetic support due to the parent’s refusal to apply anaesthesia for the MR investigation. The other two excluded patients (one 10-year old patient diagnosed with dyserythropoietic anaemia and one 16-year old patient diagnosed with acquired aplastic anaemia) could not hold their breath because of the complications of the primary disease. For all excluded patients, T2* maps obtained using the FB-mTFE sequence were suitable for T2* assessment. Figure 3 demonstrates an example of the excluded data from a 5-year old boy with Diamond–Blackfan anaemia, who underwent the study without anaesthetic support. For this patient, the images obtained using the FB-mTFE sequence could be used for T2* mapping (mean 15.5 ms, SD 1.2 ms).

**Fig. 4:**
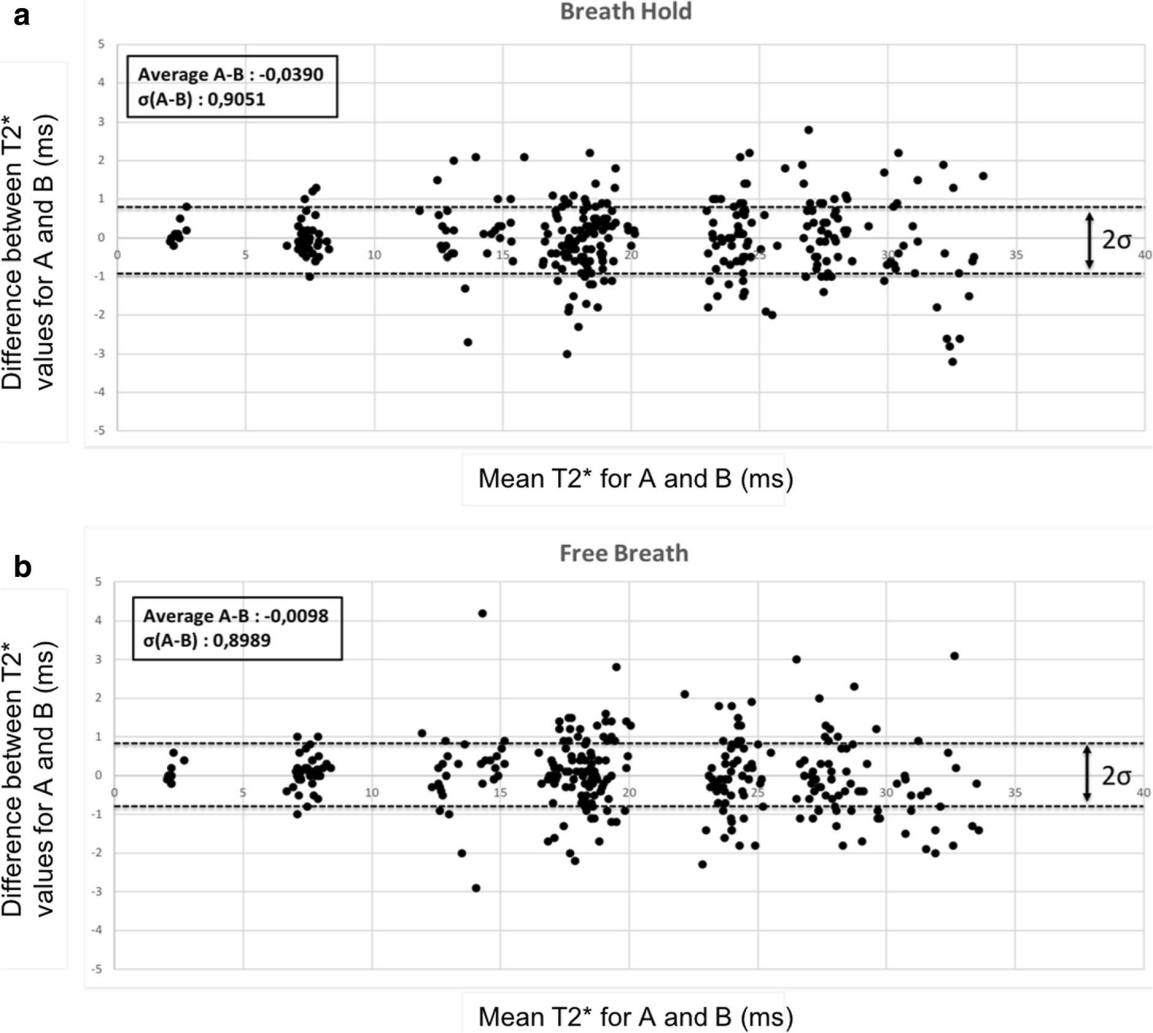
Bland–Altman plots showing the good agreement for T2* values in the interventricular septum between the first reader (**a**) and the second reader (**b**)

The coefficient of variation between the T2* values of BH-mTFE sequence and those of the FB-mTFE sequence was 3.8% for the first reader and 3.2% for the second reader. Small intra-reader data scattering confirms the accuracy of the measurements. The Bland–Altman plots showed good agreement for the breath-holding and free breathing T2* values between the two readers (Fig. 4).

The T2* values of the 32 analysed patients for the breath-holding and free-breathing sequences were normally distributed (*p* = 0.27 and *p* = 0.29 respectively). The T2* value was 19.5 ± 7.7 ms (mean ± SD) for the breath-holding sequence and 19.4 ± 7.6 ms for the free-breathing sequence, without significant difference (*p* = 0.98, Student *t* test). For the breath-holding technique, the T2* distribution in the ROI was less homogenous. For the first reader, T2* value ranged from 0.4 to 8.8 ms (SD 1.5 ms) for the breath-holding sequence, from 0.2 to 3.9 ms (SD 0.7 ms) for the free-breathing sequence; for the second reader, from 0.4 to 7.6 ms (SD 1.3 ms) and from 0.2 to 3.3 ms (SD 0.6 ms), respectively.

**Fig. 5:**
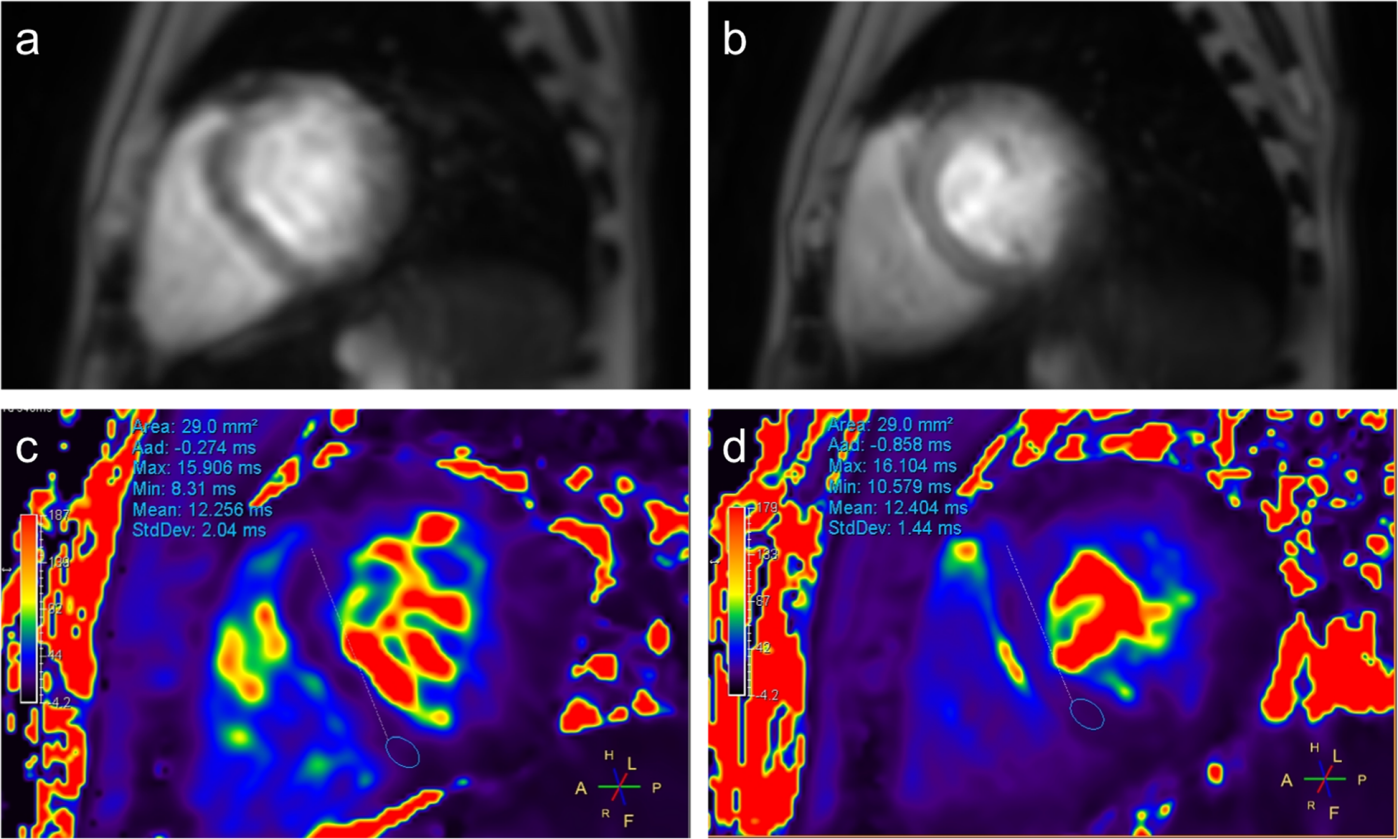
Multi-phase turbo field-echo images and T2* maps of a 9-year old boy affected with a β-thalassemia. **a** Original image obtained using the breath-holding technique. **b** Original image obtained using the free-breathing technique. **c** T2* map obtained using the breath-holding technique. **d** T2* map obtained using the free-breathing technique

**Fig. 6:**
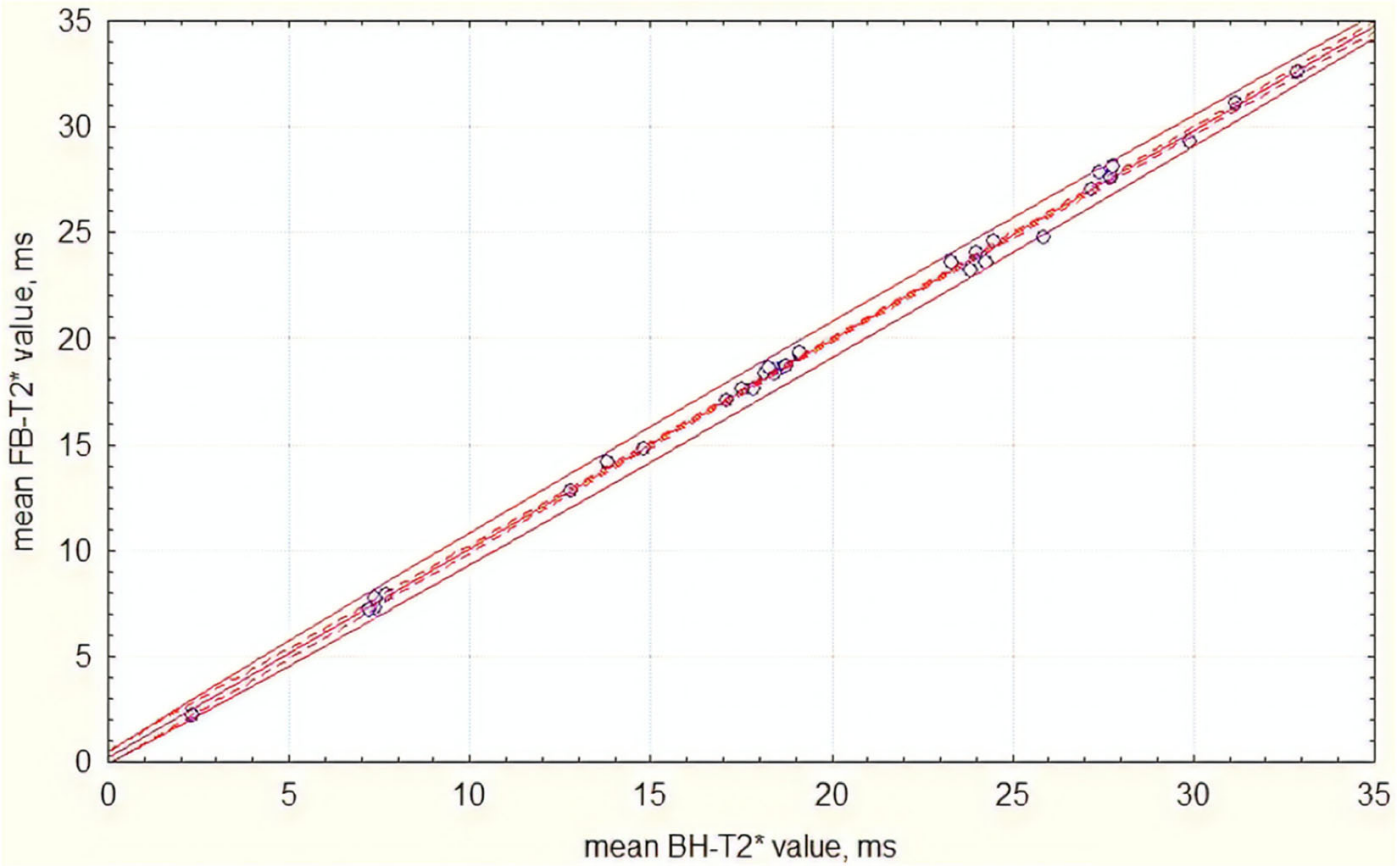
Scatterplot of T2* values obtained using free-breathing (FB) and breath-holding (BH) sequences (*n* = 32; *r* = 0.99; *R*^2^ = 0.98; *p* < 0.001). Continuous thin lines show the 95% confidence interval

Figure 5 a and b demonstrate the examples of mapping using the free-breathing and the breath-holding techniques. Comparison of the measurements showed a strong positive correlation between T2* values of the two methods (*r* = 0.99, *R*^2^ = 0.98; *p* < 0.001) (Fig. 6).

## Discussion

Reliable data related to the iron level in the myocardium can be obtained using GRE sequences and measuring the T2* value. Thus, this approach has become an important diagnostic tool for planning and monitoring the chelation therapy in patients with transfusion-dependent anaemia [26]. To this aim, breath-holding GRE sequences are commonly used [27]. However, these sequences are quite sensitive to respiratory artefacts and always require adequate breath-holding during image acquisition [28]. Thus, this standard technique is not feasible in patients who are unable to perform breath-holding commands, especially in young children.

In our study, we compared the results of the T2* mapping using standard breath-holding technique and a multiple signal accumulation free-breathing technique and found that 14% of the breath-holding studies had a non-diagnostic quality due to the presence of respiratory artefacts. On the contrary, in these patients, free-breathing sequence was of adequate quality and could be used for T2* mapping.

ECG-gated multiple signal accumulation techniques allow to compensate both respiratory activity of a patient. MR scanners with different magnetic field strengths are differently sensitive to the physiological movement, and multiple signal accumulation techniques with ECG-gating have been easily adapted to 1.5-T systems [23]. In our study, we demonstrated the application of ECG-gated multiple signal accumulation technique at 3-T. T2* values were measured in the interventricular septum for patients with good breath-holding, and a strong positive correlation was established between the T2* values obtained using the FB-mTFE and BH-mTFE sequences. Therefore, free-breathing scanning can be used for myocardial iron detection in paediatric practice at 3-T.

In general, the free-breathing multiple signal accumulation scanning technique may provide a higher SNR than a single breath-holding scan. The mean T2* values measured for the same ROI were not significantly different between the two techniques, however the SD of T2* was smaller for the free-breathing sequence than for the breath-holding sequence, corresponding to more homogenous T2* values for the free-breathing myocardial mapping. Therefore, the free-breathing myocardial mapping technique demonstrates higher image quality compared to the standard technique for patients who could not clearly follow the breath-holding instructions, especially in patients aged from 2 to 7 years. Of note, based on our experience, it is rare that patients under 7 years of age are able to methodically hold their breath for 8–12 s.

However, most of the haematologic patients from 5 to 7 years of age can lie in the MR scanner without any movement for 20–30 min. Despite the motionless of this patient group, the usage of the multi-phase GRE sequence without anaesthetic support is impossible due to the huge number of breathing artefacts, which make it difficult to generate the relaxometry maps and calculate the T2* values.

Three of the patients in the age from 5 to 7 years were scanned without anaesthetic support. For these patients, five or more re-scans (repetition of the scans) were conducted for the breath-holding T2* analysis without successful results: images were corrupted by the respiratory artefacts. The duration of the studies was extended from 15 s to 2 min including attempts and preparation time. The use of the free-breathing sequence allows to reduce the number of re-scans due to the fuzzy holding of the patient’s breath and the absence of the breath-holding preparation increases the comfort of the study.

The free-breathing protocol will help to optimise the investigation of patients aged from 2 to 7 years. Some cases will allow to omit anaesthesia or at least to reduce its depth, which is extremely important in paediatric practice. Despite the fact that the duration of the investigation by free-breathing technique with artefact compensation is longer than the standard breath-holding technique, in paediatric practice, the whole duration of the study will be reduced. In most cases, children do not manage to adequately hold breath from the first try, and the study continues with the repetition of the sequences several times. This makes it possible to consider the free-breathing T2* mapping technique potentially promising for the myocardium iron assessment also in other patients who are not able to hold their breath due to their health condition.

Our study has some limitations. This work was made only on a 3-T scanner, and we cannot discuss the performance of this technique on scanners with another field strength. On the other hand, motion artefacts on 1.5-T scanners are usually less noticeable than on 3-T scanners, which allows us to speculate that this technique could be useful also for 1.5-T scanners. In addition, only 3 out of 17 patients under 7 years of age were scanned without anaesthetic support, which does not allow us to conclude on the possibility of scanning these patients without anaesthesia, but suggests that it can be possible. Further research is needed in patients under 7 years of age.

In conclusion, the FB-mTFE sequence allowed to obtain good quality T2* myocardial maps at 3-T for patients with iron overload in different groups of haematological diseases. Free-breathing scans with multiple signal accumulation technique did not require additional research packages and also facilitated and shortened the duration of the investigation in young children under anaesthetic support and in some cases allowed to even refuse it. This approach may pave the way for scanning patients who are unable to hold their breath for medical reasons without anaesthetic support. Further studies in patients of different age ranges are required.

## Data Availability

The data can be made available upon the request.

## Abbreviations

BH-mTFE: Breath-holding multi turbo-field echo
ECG: Electrocardiography
FB-mTFE: Free-breathing multi turbo-field echo
GRE: Gradient echo
MR: Magnetic resonance
ROI: Region of interest
SD: Standard deviation
SNR: Signal-to-noise ratio
